# Dysregulated long noncoding RNAs (lncRNAs) in hepatocellular carcinoma: implications for tumorigenesis, disease progression, and liver cancer stem cells

**DOI:** 10.1186/s12943-017-0734-4

**Published:** 2017-10-23

**Authors:** Xiaoqi Huo, Shuanglin Han, Guang Wu, Olivier Latchoumanin, Gang Zhou, Lionel Hebbard, Jacob George, Liang Qiao

**Affiliations:** 1 Storr Liver Centre, Westmead Institute for Medical Research, University of Sydney and Westmead Hospital, Westmead, NSW 2145 Australia; 2 Department of Gastroenterology, The Second Affiliated Hospital of Dalian Medical University, Dalian, Liaoning Province 116027 China; 3 Department of Molecular and Cell Biology, Centre for Comparative Genomics, The Centre for Biodiscovery and Molecular Development of Therapeutics, James Cook University, Australian Institute of Tropical Health and Medicine, QLD, Townsville, 4811 Australia

**Keywords:** Hepatocellular carcinoma (HCC), Liver cancer stem cells (LCSCs), Long non-coding RNAs (lncRNAs)

## Abstract

Hepatocellular carcinoma (HCC) is one of the most common malignant tumours with a poor prognosis worldwide. While early stage tumours can be treated with curative approaches such as liver transplantation or surgical resection, these are only suitable for a minority of patients. Those with advanced stage disease are only suitable for supportive approaches and most are resistant to the conventional chemotherapy or radiotherapy. Liver cancer stem cells (LCSCs) are a small subset of cancer cells with unlimited differentiation ability and tumour forming potential. In order to develop novel therapeutic approaches for HCC, we need to understand how the cancer develops and why treatment resistance occurs. Using high-throughput sequencing techniques, a large number of dysregulated long noncoding RNAs (lncRNAs) have been identified, and some of which are closely linked to key aspects of liver cancer pathology, progression, outcomes and for the maintenance of cancer stem cell-like properties. In addition, some lncRNAs are potential biomarkers for HCC diagnosis and may serve as the therapeutic targets. This review summarizes data recently reported lncRNAs that might be critical for the maintenance of the biological properties of LCSCs.

## Background

Although more than 70% of the whole human genome is transcriptionally very active, only less than 2% of the transcripts are eventually translated into proteins [1, 2]. Long noncoding RNAs (lncRNAs) are a subclass of functional ncRNAs (tRNA and rRNA are not included in this review); they are over 200 nucleotides in size and are incapable of encoding protein [3, 4]. lncRNAs may share some characteristics of mRNAs [4]. For instance, lncRNAs are transcribed by RNA polymerase II; they are 5′ capped, equipped with 3′ polyA (polyadenylate) tail and consist of multiple exons [4, 5]. Initially, lncRNAs were thought to be junk or transcriptional noise since they are not well conserved across species and because expression levels were relatively lower compared with mRNAs [5–8]. However, recent studies suggest that lncRNAs play a key role in many biological processes such as X chromosome inactivation, cell cycle regulation, and cardiac development [4, 5, 9, 10]. lncRNAs are also involved in the development of many diseases. For example, using microarray analysis, a list of dysregulated (either up-regulated or down-regulated) lncRNAs have been identified in many tumour types such as prostate, liver, lung, and breast cancer [11–18]. Broadly, lncRNAs can be classified as either oncogenic or tumor-suppressive [19].

Hepatocellular carcinoma (HCC) is a leading cause of cancer related death worldwide [20, 21], with more than 500,000 new cases reported every year [22]. Early stage HCC can be effectively treated by liver transplantation or curative surgery, but for advanced cases, the therapeutic strategies are limited [23, 24]. Tumour recurrence and disease relapse after therapy, as well as drug resistance are the critical issues leading to poor prognosis [24, 25].

Within the liver tumour bulk, a small group of cells known as liver cancer stem cells (LCSCs) are considered to be responsible for the initiation, recurrence and drug resistance of HCC [26, 27]. How LCSCs are regulated at the molecular level is not well understood. Knowledge of the key regulators of LCSC behaviour would facilitate the development of more effective therapeutic approaches IIn this regard, increasing evidence has shown that lncRNAs may be involved in the regulation of the biological function of LCSCs. For example, multiple lncRNAs are expressed aberrantly in LCSCs compared to non-cancer stem cells [28, 29], while some lncRNAs are required for the self-renewal and tumour propagation of LCSCs [30–32], or are closely associated with the clinico-pathological features [8, 33, 34]. However, the precise function of lncRNAs in LCSCs is poorly defined. This review summarizes current understanding of the lncRNAs and their implications for HCC and LCSCs.

## Origin and classification of lncRNA

**Fig. 1:**
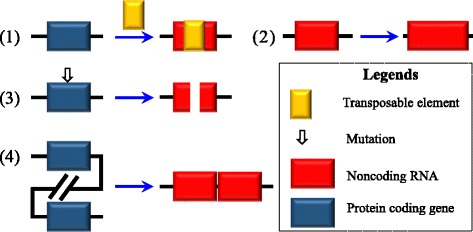
Multiple origins of lncRNAs. lncRNA may derive from (1) insertion of a transposable element; (2) duplication in non-coding RNA; (3) a previous protein coding gene; (4) chromatin rearrangement [36–38]

**Fig. 2:**
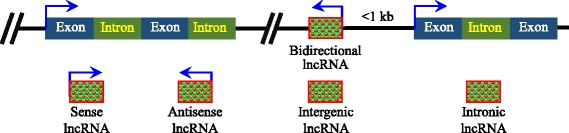
Classification of lncRNAs. Multiple type of lncRNAs have been identified, including (1) Sense lncRNA: transcribed from the sense strand of a protein coding gene; (2) Antisense lncRNA: transcribed from the antisense strand of a protein coding gene, usually overlapping the introns or exons of the sense strand; (3) Intergenic lncRNA: transcribed from the space between two protein coding genes; (4) Intronic lncRNA: incorporated inside of the introns of protein coding genes; (5) Bidirectional lncRNA: transcribed in an opposite direction with respect to the nearby protein coding gene and located within 1 kb of the promoter of the protein coding gene [8, 38]

Unlike mRNA, lncRNAs are not very well conserved across species [8, 35]. These RNAs may derive from several sources: (1) insertion of a transposable element; (2) duplication of noncoding RNA; (3) transformation from a previous protein coding gene; and (4) chromatin rearrangement [36–38] (Fig. 1). In fact, lncRNAs can be generated either from protein coding genes or non-protein coding genes. A common way of categorizing lncRNAs is based on the relative position with respect to the closest protein coding gene in the genome. In this way, lncRNAs can be classified into the following five categories: (1) intergenic lncRNAs: transcribed from the space between two protein coding genes; (2) intronic lncRNAs: incorporated inside the intron of a protein coding gene; (3) sense lncRNAs: transcribed from the sense strand of a protein coding gene; (4) antisense lncRNAs: transcribed from the antisense strand of a protein coding gene, usually overlapping the introns or exons of the sense strand; and (5) bidirectional lncRNAs: transcribed in an opposite direction with respect to the nearby protein coding gene and located within 1 kb from the promoter of the protein coding gene [8, 38] (Fig. 2).

## lncRNAs are involved in HCC through multiple mechanisms

**Fig. 3:**
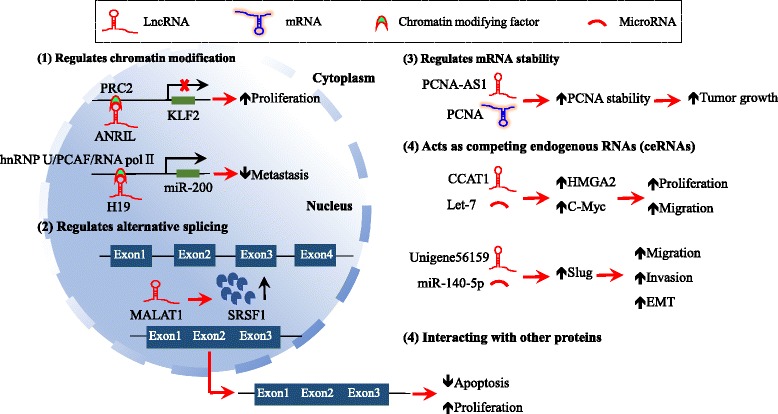
Mechanism of lncRNA function. lncRNAs are involved in HCC development through multiple mechanisms. (1) At the epigenetic level, lncRNAs can regulate the expression of target genes via recruiting chromatin modifying factors to alter chromatin structure [42]; (2) lncRNAs can control the process of alternative splicing [41]; (3) lncRNAs can regulate mRNA stability [41]; (4) lncRNAs can act as competing endogenous RNAs (ceRNAs) [47]

lncRNAs are present in either nucleus or cytoplasm, and they can interact with DNA, RNA or protein [39, 40]. Through this, they modulate the expression and stability of their downstream targets at epigenetic, transcriptional and post-transcriptional levels [41] (Fig. 3).

At the epigenetic level, lncRNAs can regulate the expression of target gene via recruiting chromatin modifying factors to alter chromatin structure [42]. For example, an up-regulated lncRNA CDKN2B antisense RNA 1 (ANRIL) in HCC has been demonstrated to promote cell proliferation; its expression level is positively associated with the Barcelona Clinic Liver Cancer (BCLC) stage [43]. This ncRNA is mostly present in the nucleus where it silences the function of Kruppel-like factor 2 (KLF2) through recruiting polycomb repressive complex 2 (PRC2) to the promoter region of KLF2 resulting in H3K27 trimethylation and KLF2 silencing [43].

lncRNAs are able to modulate the expression of non-protein coding genes such as microRNAs. In this aspect, down-regulation of a lncRNA H19 has been shown to play an important role in HCC metastasis [44]. H19 directly binds hnRNP U/PCAF/RNA polII complex resulting in the active transcription of the miR-200 family by acetylating histone H3 of its promoter [44].

lncRNAs have also been found to function at a post-transcriptional level to control the progression of HCC via regulating alternative splicing and mRNA stability [41]. For example, an up-regulated lncRNA termed metastasis-associated lung adenocarcinoma transcript 1 (MALAT1) in HCC increases the expression of splicing factor SRSF1 (serine and arginine rich splicing factor 1) through activating Wnt pathway, causing dys-regulated apoptosis and alternative splicing of S6 kinase1 (S6 K1), which further activates the mTOR pathway, and ultimately resulting in HCC progression [45].

Antisense lncRNAs can affect the stability of mRNAs. For example, an up-regulated antisense lncRNA termed PCNA antisense RNA 1 (PCNA-AS1) in HCC has been shown to prompt tumor growth by forming a RNA duplex with proliferating cell nuclear antigen (PCNA), leading to alteration of PCNA structure and increase in its stability [46].

Studies over the recent years have also revealed that lncRNAs can act as competing endogenous RNAs (ceRNAs) and play important roles in HCC [47]. In this perspective, an over-expressed lncRNA in HCC termed colon cancer associated transcript 1 (CCAT1) was found to competitively bind to let-7 (as evidenced by increased expression of let-7 target genes including high mobility group AT-hook2 and c-Myc), leading to increased proliferation and migration of HCC cells [48]. Similarly, a newly identified lncRNA unigene56159 also functions as a ceRNA [49]. Unigene56159 is significantly up-regulated in HBV-related HCC tissues, and is required for epithelial-mesenchymal transition (EMT) and migration of HCC cells by acting as a ceRNA for miR-140-5p to increase the expression of Slug [49].

## HCC associated lncRNA

By microarray or next-generation sequencing, a large number of significantly dysregulated lncRNAs have been identified in HCC tumour tissues as compared with adjacent non-tumorous liver [50]. Below, we will summarize some of the deregulated lncRNAs that may play key roles in HCC development and progression, and describe their correlation with the clinical outcomes of HCC. The reported data may provide new sights for the early diagnosis and for therapeutic targeting.

## Dysregulated lncRNAs implicated in the regulation of HCC proliferation

Deregulated control of the ability to proliferate and undergo apoptosis is an important feature of cancers [51]. Many lncRNAs are involved in the regulation of uncontrolled cell growth in HCC. TP73 antisense RNA 1 (TP73-AS1) is a lncRNA that is overexpressed in HCC tissues compared to the adjacent non-tumorous liver [52]. TP73-AS1 competes with high mobility group box 1 (HMGB1) to bind miR200a and activates HMGB1/RAGE axis thereby promoting the proliferation of HCC cells [52]. As such, knockdown of TP73-AS1 by siRNA leads to impaired proliferation and colony formation of HepG2 and HCCLM3 cells [52].

Colon cancer associated transcript 2 (CCAT2) is an oncogenic lncRNA transcribed from chromosome 8q24.21 [53]. Up-regulation of CCAT2 has been identified in several HCC cell lines (HepG2, Hep3B, HCCLM3 and Huh7) and tumour tissues [53]. Overexpression of CCAT2 in HepG2 and Huh7 cells significantly promotes their proliferation while apoptosis is reduced [53]. Opposite results were observed when CCAT2 was knocked down in these cells [53].

lncRNAs may perform their biological functions via interacting with other proteins. For example, overexpression of a lncRNA termed ultraconserved element 338 (uc.338) was shown to promote the proliferation of HCC cells in a BMI1-dependent manner as confirmed by RNA pull down and RNA immunoprecipitation assays [54].

In addition to up-regulated lncRNAs, some significantly down-regulated lncRNAs play critical roles in HCC. For example, significantly reduced expression of lncRNA BRAF-activated noncoding RNA (BANCR) has been observed in HCC tissues and HCC cell lines (Huh7, HepG2 and SMMC-7721). Consequently, forced overexpression of lncRNA BANCR in these cells inhibits their proliferation in vitro and in vivo [55]. Another down-regulated lncRNA termed long noncoding RNA FTX (lnc-FTX) in HCC can modulate the proliferation of HCC and is closely related with the prognosis [56]. Thus, lnc-FTX itself functions as a tumor suppressor by interacting with minichromosome maintenance protein 2 (MCM2), suppressing the proliferation of HCC cells. In addition, lnc-FTX may function as a ceRNA by binding to miR-374a, thereby inhibiting the activity of Wnt pathway and decrease in EMT and invasion [56]. Patients with low hepatic lnc-FTX have reduced survival and early recurrence suggesting that lnc-FTX might serve as a biomarker of prognosis [56].

## LncRNAs involved in the regulation of epithelial to mesenchymal transition in HCC

Previous studies have confirmed that epithelial to mesenchymal transition (EMT) contributes to multiple stages of cancer development such as progression, invasion and metastasis [57]. Increasing evidence now demonstrates that many lncRNAs play a role in triggering EMT during the pathogenesis of HCC. For instance, up-regulation of a lncRNA urithelial carcinoma-associated 1 (UCA1) in HCC tissues has been closely linked to tumour size, invasion and tumour progression [58]. In vitro studies also suggested significant up-regulation of lncRNA UCA1 in multiple HCC cell lines (Huh7, SK-Hep-1, MHCC97H and MHCC97L) [58]. By using gain- and lose-of-function analysis in MHCC97H and MHCC97L cells, a positive correlation between the expression of lncRNA UCA1 and EMT markers such as Snail2 and Vimentin was identified, as well as a negative correlation between the expression of lncRNA UCA1 and E-cadherin [58]. These data suggest that overexpression of lncRNA UCA1 may promote HCC development by driving EMT [58]. As mentioned above, CCAT2 is a lncRNA up-regulated in HCC and high level of lncRNA CCAT2 correlates with poorer patient survival and a worse prognosis [59]. This lncRNA not only promotes the proliferation of HCC cells, but also activates EMT via up-regulating Snail2 [59]. Similarly, two other lncRNAs Sprouty4-Intron 1 (SPRY4-IT1) and small nucleolar RNA host gene 20 (SNHG20) that are overexpressed in HCC have been shown to prompt EMT via up-regulating the transcription factors twist1 and Vimentin while reducing the expression of E-cadherin [60–62].

## Role of lncRNAs in HCC drug resistance

Resistance to chemotherapy or radiotherapy is a serious hurdle in the clinical management of patients with HCC. Indeed, resistance to previously first-line anticancer agents like adriamycin is responsible for a large portion of treatment failure in patients with advanced liver cancer [63–65]. Multiple studies have revealed that some lncRNAs may be mechanistically responsible for the development of anticancer drug resistance in HCC.

Taurine up-regulated gene 1 (TUG1) is an overexpressed lncRNA present in adriamycin-resistant HCC tissues and cell lines (SMMC-7721/ADM and HepG2/ADM). In turn, siTUG1 mediated knockdown of TUG1 restored the sensitivity of resistant cells to adriamycin [65]. Mechanistically, TUG1 was found to regulate the expression of P-glycoprotein (P-gp) and multidrug-resistance 1 (MDR1) which are closely related to the development of drug resistance [65].

Although the mechanism of drug resistance is not well understood, protective autophagy has been proposed to be involved in chemoresistance [66, 67]. Highly up-regulated in liver cancer (HULC) RNA is another commonly up-regulated lncRNA in HCC [67]. Up-regulation of HULC has been linked to the development of resistance of HCC cells to oxaliplatin, 5-fluorouracil (5-FU) and pirarubicin (THP) via activating autophagy by protecting the degradation of silent information regulator 1 (SIRT1) [67]. This finding suggests that HULC may be a novel target for improving the chemosensitivity [67].

## Role of lncRNAs in the regulation of liver cancer stem cells (LCSCs)

Although LCSCs only represent a small portion of cells within HCCs, they are a critical subset of cells that are believed to account for the initiation, progression, recurrence, metastasis and treatment resistance of liver cancer [26, 27]. An increasing number of dysregulated lncRNAs have been identified in LCSCs. Among them, a range of lncRNAs directly regulate the stem cell features or indirectly regulate LCSCs via interacting with other key signaling pathways, such as Wnt and IL6/STAT3 pathways [68]. Some identified lncRNAs involved in the regulation of the function of LCSCs are summarized below.

### HOX antisense intergenic RNA (HOTAIR)

HOX antisense intergenic RNA (HOTAIR) is a 2.2 kb lncRNA localized to chromosome 12q13.13 within Homeobox C (HOXC) cluster [69, 70]. The oncogenic role of HOTAIR has been demonstrated in multiple cancers such as breast cancer, melanoma, and colorectal cancer [19, 71, 72]. Up-regulation of HOTAIR has also been identified in HCC [73] and LCSCs where it has been linked to the expansion of stem cells. Mechanistically, HOTAIR reduces the recruitment of P300, RNA polymerase II (RNApol II) and cAMP responsive element binding protein 1 (CREB) to the promoter of SET Domain-Containing Protein 2 (SETD2) thereby suppressing SETD2 activity [73]. Reduced SETD2 activity impairs the binding to histone 3 (H3) and subsequently leads to reduced trimethylation on histone H3 thirty-sixth lysine (H3K36me3), resulting in reduced formation of the H3K36me-hMSH2-hMSH6-SKP2 complex. These changes eventually disrupt DNA damage repair mechanisms and cause microsatellite instability (MSI) [73].

### Lnc-DILC

Lnc-DILC is a recently identified novel lncRNA with tumor suppressive activity [28]. By microarray and qRT-PCR analysis, reduced expression of lnc-DILC has been observed in LCSCs [28]. Using RNA in situ hybridization, lnc-DILC was found to reside in both the nucleus and cytoplasm [28]. In vivo and in vitro experiments demonstrate that lnc-DILC suppresses the expansion of LCSCs via inhibiting interleukin 6 (IL-6)/JAK2/STAT3 signaling [28]. Using multivariate analysis, it was found that lnc-DILC may serve as an independent prognostic marker for HCC. Thus, patients with low hepatic expression have shorter survival time and a higher frequency of tumour recurrence than those with higher lnc-DILC levels [28]. These data indicate that lnc-DILC could serve as a potential target for HCC therapy and as a biomarker for monitoring outcome.

### LncBRM

LINCR-0003 (lncBRM) is a lncRNA overexpressed in HCC tissues and LCSCs; it is required for the maintenance of the stemness features and for the biological properties of LCSCs through Yes-associated protein 1 (YAP1) signaling [29]. LncBRM is present in the cell nucleus and is localized between actin beta like 2 (ACTBL2) and polo like kinase 2 (PLK2) on chromosome 5, with a size of 1321 nt containing six exons [29]. By RNA pulldown assay, lncBRM interactt with BRM and this interaction facilitates the formation of BRG1-embedded BAF (BRG1-associated factor) complex. The latter can then bind to the promoter region of YAP1 and along with the enrichment of KLF4, ultimately leads to activation of YAP1 signaling, a critical pathway for the maintenance of stemness features of LCSCs [29].

### Cancer up-regulated drug resistant (CUDR)

Cancer up-regulated drug resistant (CUDR) is an up-regulated oncogenic lncRNA in LCSCs [74]. CUDR is present in both the nucleus and cytoplasm and exerts its biological function by promoting the growth of LCSCs through up-regulating the expression of CyclinD1 and down-regulating phosphatase and tensin homolog (PTEN). This promotes the formation of a CUDR-CyclinD1 complex [74] that inhibits the methylation of H19 promoter, increases H19 transcription and increases the formation of telomerase reverse transcriptase-telomerase RNA component (TERT-TERC) complex, thereby leading to increased telomerase activity [74]. In addition, the CUDR-CyclinD1 complex can be recruited by CCCTC-binding factor (CTCF) to form a CUDR-CyclinD1-CTCF complex which can engage the promoter of c-Myc leading to increased c-Myc expression [74]. These changes synergistically contribute to the expansion of LCSCs [74].

### LncTCF7

Wnt signaling has been confirmed to play an important role in the self-renewal and propagation of CSCs [75]. Recently, an intergenic lncRNA termed lncTCF7 was found to activate the Wnt pathway in LCSCs by modulating its nearby gene expression [30]. lncTCF7 is present in the nucleus and is up-regulated in HCCs and LCSCs [30]. lncTCF7 sustains the self-renewal of LCSCs by recruiting SWItch/Sucrose Non-Fermentable (SWI/SNF) complex to the promoter of its nearby protein coding gene transcription factor 7 (TCF7) leading to increased TCF7 transcription and activation of Wnt signaling pathway [30]. Of note, lncTCF7 can barely be detectable in normal liver, making it a promising biomarker for the diagnosis and therapeutic targeting of liver cancer [30].

### LncRNA for β-catenin methylation (lnc-β-Catm)

lncRNA for β-catenin methylation (lnc-β-Catm) is an overexpressed lncRNA identified in LCSCs [31]. lnc-β-Catm is localized to the chromosome 1q between interferon regulatory factor 2 binding protein 2 (IRF2BP2) and translocase of outer mitochondrial membrane 20 (TOMM20) genes with a size of 2281 nt and contains two exons [31]. lnc-β-Catm regulates the self-renewal of LCSCs via activating the Wnt signaling [31]. lnc-β-Catm mediates the formation of a RNA-protein complex consisting of β-catenin and enhancer of zeste homolog 2 (EZH2) in which β-catenin is methylated by EZH2 [31]. This leads to increased stability of β-catenin and eventually triggers activation of Wnt signaling pathway [31]. Patients with high levels of lnc-β-Catm have a relatively poorer prognosis [31].

### LncRNA *calmodulin binding transcription activator 1 (*CAMTA1)

lncRNA CAMTA1 is highly expressed in HCCs and LCSCs [32]. Using gain- and loss-of-function studies, it was found that lncRNA CAMTA1 was essential for the maintenance of the CSC properties in HCC through regulating the expression of calmodulin binding transcription activator 1 (CAMTA1) [32]. Direct binding of lncCAMTA1 to the CAMTA1 promoter changes the chromatin structure and inhibits the transcription of CAMTA1 as confirmed by chromatin isolation by the RNA purification Assay (CHIRP) [32]. Inhibition of CAMTA1 facilitates the maintenance of cancer stem cell-like properties [32]. The essential oncogenic role of lncRNA CAMTA1 in liver cancer was further supported by the finding that patients with higher levels of lncRNA CAMTA1 have shorter survival and an increased rate of early recurrence [32].

### LncRNA DANCR

lncRNA differentiation antagonizing non-protein coding RNA (DANCR) is a lncRNA with significant up-regulation in HCC and LCSCs [76, 77]. DNACR is involved in the maintenance and regulation of the stemness feature of LCSCs [77]. More mechanistic studies have shown that DNACR promotes HCC via acting as a ceRNA since it competitively impairs the binding between CTNNB1 (β-catenin) and miRNA (miR-214, miR-320a and miR-199a), leading to increased CTNNB1 stability [77]. Patients with DANCR overexpression often show increased rates of tumour recurrence and poor survival [77].

### Plasmacytoma variant translocation 1 (PVT1)

lncRNA plasmacytoma variant translocation 1 (PVT1) is an oncofetal lncRNA with increased expression and an oncogenic role in many solid tumours (e.g., cervical, gastric, colorectal, esophageal, pancreatic, non-small cell lung, ovarian, bladder and thyroid cancers as well as in acute promyelocytic leukemia) [78–87]. Overexpression of lncPVT1 has been found not only in pre-cancerous liver conditions such as inactivated hepatic stellate cells (HSCs) and fibrotic liver tissues [88], but also in adult HCC [89]. In the development of liver fibrosis, lncPVT1 competitively binds to miR-152 to promote liver fibrosis via the Hedgehog (Hh) and EMT pathway [90]. In the development of liver cancer, both in vivo and in vitro studies demonstrate that lncPVT1 can prompt the proliferation and stem cell-like features of liver cancer cells by stabilizing an RNA binding protein named nucleolar protein (NOP2) [89]. Clinically, lncPVT1 is positively associated with tumour size, tumour stage and HBV infection [89].

### Clinical implications of lncRNAs in the early diagnosis and survival prediction

Early diagnosis and intervention remains the most effective approach to achieving a better outcome for HCC [91]. In this aspect, sensitive and specific markers for early diagnosis are urgently required. Alpha fetal protein (AFP) has been widely used as a diagnostic marker for HCC, but its sensitivity and specificity are suboptimal [92, 93]. lncRNAs with a significantly aberrant expression profile in HCC tissues as opposed to healthy liver may hold great potential as diagnostic markers [94]. Some lncRNAs may be stably present in body fluids such as plasma and their expression levels are consistently associated with the progression of HCC [94]. These lncRNAs hold potential value as diagnostic markers for HCC [94].

HULC is an up-regulated lncRNA in HCC. Meanwhile, high levels of HULC has been detected in the plasma of HCC patients with higher Edmondson grades. Hence, it was proposed that HULC may possess diagnostic value [95]. Through screening blood samples of HCC patients by microarray, an expression profile of circulating lncRNAs has been established [96]. By qRT-PCR assay, three novel lncRNAs (RP11-160H22.5, XLOC-014172 and LOC149086) were shown to be significantly up-regulated in liver and matched plasma from HCC patients [96]. Furthermore, increased expression of XLOC-014172 and LOC149086 was found to be predictors for HCC metastasis [96].

The above lncRNAs are stably present in patient serum and possess moderate specificity (73%) and sensitivity (82%) for HCC [96]. Hence, they have potential either alone or more likely in combination with other variables as biomarkers for diagnosis and monitoring.

## Prospective and conclusion

With the application of high-throughput sequencing, a tremendous number of newly identified dysregulated lncRNAs are reported every year. At present, more than 30,000 dysregulated lncRNAs have been identified in humans and the number is still increasing as a result of improved screening technology [8, 97]. Of the large number of lncRNAs identified so far, some (e.g., TUG1 and HOTAIR) have been shown to play very important roles in multiple aspects of liver cancer [65, 73] such as tumourigenesis, tumour recurrence and drug resistance (Table. 1). Some lncRNAs (e.g., lncBRM, lncTCF7 and PVT1) play regulatory roles in the maintenance of stemness properties of LCSCs and hence may have a role in tumour initiation [29, 30, 89] (Table. 1). Other lncRNAs (e.g., HULC) have potential to serve as diagnostic markers [95]. However, detailed clarification of the roles of lncRNAs in liver cancer is lacking, partially because lncRNAs have more complicated functions and multiple modes of functions [98]. Besides, unlike mRNAs, lncRNAs are not conserved across the species making it difficult for translating the data from the animal models to humans [35].

**Table 1:** Deregulated lncRNAs in HCC

	lncRNAs	Function	Mechanisms	References
Up-regulated	ANRIL	Promotes cell proliferation	Interacts with PRC2 to silent KLF2	[43]
	MALAT1	Induces apoptosis, activates mTOR pathway	Increases the splicing factor SRSF1	[45]
	PCNA-AS1	Promotes tumour growth	Forms a duplex with PCNA, increases PCNA stability	[46]
	CCAT1	Promotes cell proliferation and migration	Binds let-7 as a ceRNA	[48]
	Unigene56159	Induces EMT and migration	Binds miR-140-5p as a ceRNA	[49]
	TP73-AS1	Promotes cell proliferation	Competes with HMGB1 to bind miR200a, activate HMGB1/RAGE axis	[52]
	CCAT2	Promote cell proliferation, EMT and reduces apoptosis	Up-regulates Snail2	[53, 59]
	uc.338	Promotes cell proliferation	Interacts with BMI1	[54]
	UCA1	Promotes invasion and EMT	Interacts with miR-203, regulates the expression of Snail2	[58]
	SPRY4-IT1	Promotes EMT	Up-regulates twist1 and Vimentin, reduces the expression of E-cadherin	[60]
	SNHG20	Promotes EMT	Up-regulates twist1 and Vimentin, reduces the expression of E-cadherin	[61, 62]
	TUG1	Induces drug resistance	Regulates the expression of P-gp and MDR1	[65]
	HULC	Induces resistance to oxaliplatin, 5-FU and THP	Activates autophagy	[67]
	HOTAIR	Damages DNA repair and induces MSI	Reduces the recruitment of P300, RNA Pol II and CREB to SETD2 promoter	[73]
	lncBRM	Maintains stemness and properities of LCSCs	Interacts with BRM and activates YAP1 signalling	[29]
	CUDR	Promotes growth of LCSCs	Up-regulates CyclinD1, down-regulated PTEN	[74]
	lncTCF7	Promotes self-renewal and propagation of CSCs	Recruits SWI/SNF complex, activates Wnt pathway	[30]
	lnc-β-catenin	Regulates self-renewal of LCSCs	Mediates the formation of β-catenin -EZH2 complex, activates Wnt signalling	[31]
	lncRNA CAMTA1	Maintains CSC properties	Regulates the expression of CAMTA1	[32]
	lncRNA DANCR	Maintains stemness of LCSCs	Impairs interaction between CTNNB1 and miRNA as a ceRNA	[77]
	PVT1	Promotes cell proliferation and stem cell-like features	Stabilizes NOP2	[89]
Down-regulated	H19	Mediates metastasis of HCC	Binds hnRNP U/PCAF/RNA Pol II complex to increase the expression of miR-200 family	[44]
	BANCR	Promotes cell proliferation		[55]
	lnc-FTX	Modulate cell proliferation	Interacts with MCM2 and binds miR-374a as a ceRNA	[56]
	lnc-DILC	Promotes expansion of LCSCs	Inhibits IL-6/JAK2/STAT3	[28]

In terms of clinical applications, although some lncRNAs (e.g.,HULC) have shown their potential in the early diagnosis and prognosis prediction for patients with liver cancer [95], large scale clinical studies in liver cancer patients with various etiologies are needed to further validate their efficacy, and more studies are needed to explore the lncRNAs that might serve as the more specific and sensitive markers for the diagnosis and therapy for liver cancer.

## Abbreviations

5-FU: 5-fluorouracil
ACTBL2: actin beta like 2
AFP: alpha fetal protein
ANRIL: CDKN2B antisense RNA 1
BAF: BRG1-associated factor
BANCR: BRAF-activated noncoding RNA
BCLC: Barcelona Clinic Liver Cancer
CAMTA1: calmodulin binding transcription activator 1
CCAT1: colon cancer associated transcript 1
CCAT2: colon cancer associated transcript 2
ceRNA: competing endogenous RNAs
CHIRP: chromatin isolation by RNA purification Assay
CREB: cAMP responsive element binding protein 1
CTCF: CCCTC-binding factor
CUDR: cancer up-regulated drug resistant
DANCR: differentiation antagonizing non-protein coding RNA
EMT: epithelial-mesenchymal transition
EZH2: enhancer of zeste homolog 2
H3: histone 3
H3K36me3: histone H3 thirty-sixth lysine
HCC: Hepatocellular carcinoma
Hh: Hedgehog
HMGB1: high mobility group box 1
HOTAIR: HOX antisense intergenic RNA
HOXC: Homeobox C
HSCs: hepatic stellate cells
HULC: highly upregulated in liver cancer
IL-6: interleukin 6
IRF2BP2: interferon regulatory factor 2 binding protein 2
KLF2: Kruppel-like factor 2
LCSCs: Liver cancer stem cells
lncRNAs: long noncoding RNAs
lnc-β-Catm: lncRNA for β-catenin methylation
MALAT1: metastasis-associated lung adenocarcinoma transcript 1
MCM2: minichromosome maintenance protein 2
MDR1: multidrug-resistance 1
MSI: microsatellite instability
NOP2: nucleolar protein
PCNA: proliferating cell nuclear antigen
PCNA-AS1: PCNA antisense RNA 1
P-gp: P-glycoprotein
PLK2: polo like kinase 2
PRC2: polycomb repressive complex 2
PTEN: phosphatase and tensin homolog
PVT1: plasmacytoma variant translocation 1
RNApol II: RNA polymerase II
S6 K1: S6 kinase 1
SETD2: SET Domain-Containing Protein 2
SIRT1: silent information regulator 1
SNHG20: small nucleolar RNA host gene 20
SPRY4-IT1: Sprouty4-Intron 1
SRSF1: serine and arginine rich splicing factor 1
SWI/SNF: SWItch/Sucrose Non-Fermentable
TCF7: transcription factor 7
TERT-TERC: telomerase reverse transcriptase-telomerase RNA component
THP: pirarubicin
TOMM20: translocase of outer mitochondrial membrane 20
TP73-AS1: TP73 antisense RNA 1
TUG1: Taurine up-regulated gene 1
UCA1: urithelial carcinoma-associated 1
YAP1: Yes-associated protein 1
